# Measurement Feedback System for Intensive Neurorehabilitation after Severe Acquired Brain Injury

**DOI:** 10.1007/s10916-022-01809-z

**Published:** 2022-04-04

**Authors:** Ruud van der Veen, Jaap Oosterlaan, Mike Bos, Mara van Dooren, Işıl Düdükçü, Andries van Iperen, Linda Kooiman, Karel Nicolas, Saskia Peerdeman, Marsh Königs

**Affiliations:** 1 Emma Neuroscience Group, Department of Pediatrics, Amsterdam Reproduction & Development, Emma Children’s Hospital, Amsterdam UMC, University of Amsterdam, Meibergdreef 9, 1105 AZ Amsterdam, Netherlands; 2 Daan Theeuwes Center for Intensive Neurorehabilitation, Woerden, Netherlands; 3 Department of Neurosurgery, Amsterdam UMC, Vrije Universiteit Amsterdam, Amsterdam, Netherlands

**Keywords:** Measurement feedback system (MFS), Implementation, Precision medicine, Acquired brain injury

## Abstract

Outcome of acquired brain injury (ABI) and the potential for neurorehabilitation are subject to distinct heterogeneity between patients. Limited knowledge of the complex constellation of determinants at play interferes with the possibility to deploy precision medicine in neurorehabilitation. Measurement Feedback Systems (MFS) structure clinical data collection and deliver the measurement results as feedback to clinicians, thereby facilitating progress monitoring, promoting balanced patient-centered discussion and shared decision making. Accumulation of clinical data in the MFS also enables data-driven precision rehabilitation medicine. This article describes the development and implementation of a MFS for neurorehabilitation after ABI. The MFS consists of specialized measurement tracks which are developed together with representatives of each discipline in the multidisciplinary team. The MFS is built into a digital platform that automatically distributes measurements among clinicians, at predetermined time points during the inpatient treatment, outpatient treatment and follow-up. The results of all measurements are visualized in individual patient dashboards that are accessible for all clinicians involved in treatment. Since step-wise implementation, 124 patients have been registered on the MFS platform so far, providing an average of more than 200 new measurements per week. Currently, more than 15,000 clinical measurements are captured in the MFS. The current overall completion rate of measurements is 86,4%. This study shows that structured clinical assessment and feedback is feasible in the context of neurorehabilitation after severe ABI. The future directions are discussed for MFS data in our Health Intelligence Program, which aims at periodic care evaluation and the transition of neurorehabilitation care towards precision medicine.

**Supplementary information:**

The online version contains supplementary material available at 10.1007/s10916-022-01809-z.

## Background

Worldwide, an estimated 85 million individuals suffer from cerebrovascular accidents and traumatic brain injury (TBI) annually, [1, 2] representing the most prevalent causes of acquired brain injury (ABI) [3]. ABI can cause prominent and persisting impairments in a range of function domains (e.g. physical, neurocognitive and behavioral functioning) [4] negatively impacting on participation [5] and quality of life [6]. Following acute treatment in the trauma center, multidisciplinary neurorehabilitation is typically indicated for patients with ABI and impairments in multiple function domains [7]. Neurorehabilitation treatment aims to promote recovery, limit the degree and impact of impairment and maximize participation and quality of life. However, outcome of neurorehabilitation is subject to distinct heterogeneity between patients with ABI [8]. 

The heterogeneity between patient outcomes is likely to be fueled by complex interaction between a wide range of determinants, such as premorbid patient characteristics [9], ABI etiology [10], acute manifestation of ABI [11], treatment characteristics [12] and environmental factors. [13]. The complexity of factors that determine outcome has impeded the ability to (1) determine the exact expected treatment response (‘rehabilitation potential’ [14]); (2) provide a reliable prognosis of outcome; (3) personalize the treatment to optimize the treatment response at the individual level; and (4) determine the moment in time at which treatment should be halted as no further improvements in outcome can reasonably be expected. The current situation urges for better understanding of determinants of outcome in ABI [15, 16].

Precision medicine is an emerging approach that takes into account a multitude of determinants to customize health care towards the patient’s individual needs, ultimately aiming to optimize outcome [17]. The concept of precision medicine is highly dependent on the availability of data that can be used to model the complex relations between determinants and outcome [18]. Currently, decision-making in the field of neurorehabilitation still largely relies on subjective information [14, 19, 20]. Clinical data is often not collected systematically, and neurorehabilitation settings typically widely vary in the use of measurement instruments and timing of assessment, primarily based on the preference of the clinician. Moreover, clinical data is often not registered in an easily accessible and re-usable manner (e.g. in a database). These factors affect the value of clinical data, while also severely limiting its potential for use in precision medicine in neurorehabilitation. Structured clinical data collection and registration could make a significant contribution to the field of neurorehabilitation. According to qualitative research on interdisciplinary rehabilitation team meetings, the use of structured clinical data can promote a shared understanding of the patients' functioning and provide a common ground for balanced patient-centered discussion [21]. Structured clinical data can also help to improve the composition of individual treatment plans, monitoring of progress over time and determination of the treatment response [21]. Measurement feedback systems (MFS) originate from mental health care and comprise structured measurements that track treatment progress, including deliverance of the measurement results to clinicians as timely and clinically useful feedback [22]. Thereby, MFS is a compelling method for structuring clinical data in neurorehabilitation, while simultaneously accumulating a valuable database for the transition towards precision medicine. Nevertheless, implementation of structural measurements in clinical practice has proven to be challenging [23–25]. Known barriers can be attributed to a lack of agreement on the standardization of instruments, the availability of information technology systems, lack of time and resources, insufficient compliance of clinicians and/or patients, and lack of knowledge [26]. Therefore, successful implementation of MFS requires a coordinated effort at the individual, team and management levels of an organization [27].

This study describes the development and implementation of a MFS for neurorehabilitation. The MFS is considered to directly improve clinical care by: (1) collectively designing a MFS that unifies the methodology and timing of clinical assessments based on multidisciplinary consensus; (2) improving individual progress monitoring and evaluation by providing easy access to data visualizations in individual discipline-specific patient dashboards; (3) facilitating interdisciplinary clinical decision making through easy cross-discipline access to patient dashboards; (4) improving patient education and shared decision making through the availability of comprehensive progress monitoring data. Importantly, accumulation of clinical data in the MFS on the group level is considered to contribute to continuous care evaluation and innovation and the shift towards precision medicine.

## Methods

### Setting

The Daan Theeuwes Center for Intensive Neurorehabilitation in Woerden, The Netherlands, is a specialized neurorehabilitation center for adolescents and young adults (16 to 35 years) with severe acquired brain injury (i.e. TBI: 65%, stroke: 25%, other: 10%). The center offers intensive rehabilitation to admitted inpatients and outpatients. For admission, patients need to be medically stable and sufficiently conscious for the rehabilitation program (a Post-Acute Level of Consciousness Scale[28] score of 8). The program consists of a particularly intensive interdisciplinary treatment (20–25 h per week) by a team consisting of a physical medicine and rehabilitation physician, case manager, neuropsychologist, counselor, physical therapist, occupational therapist, speech therapist and social worker.

### Measurement feedback system development and implementation

**Fig. 1:**
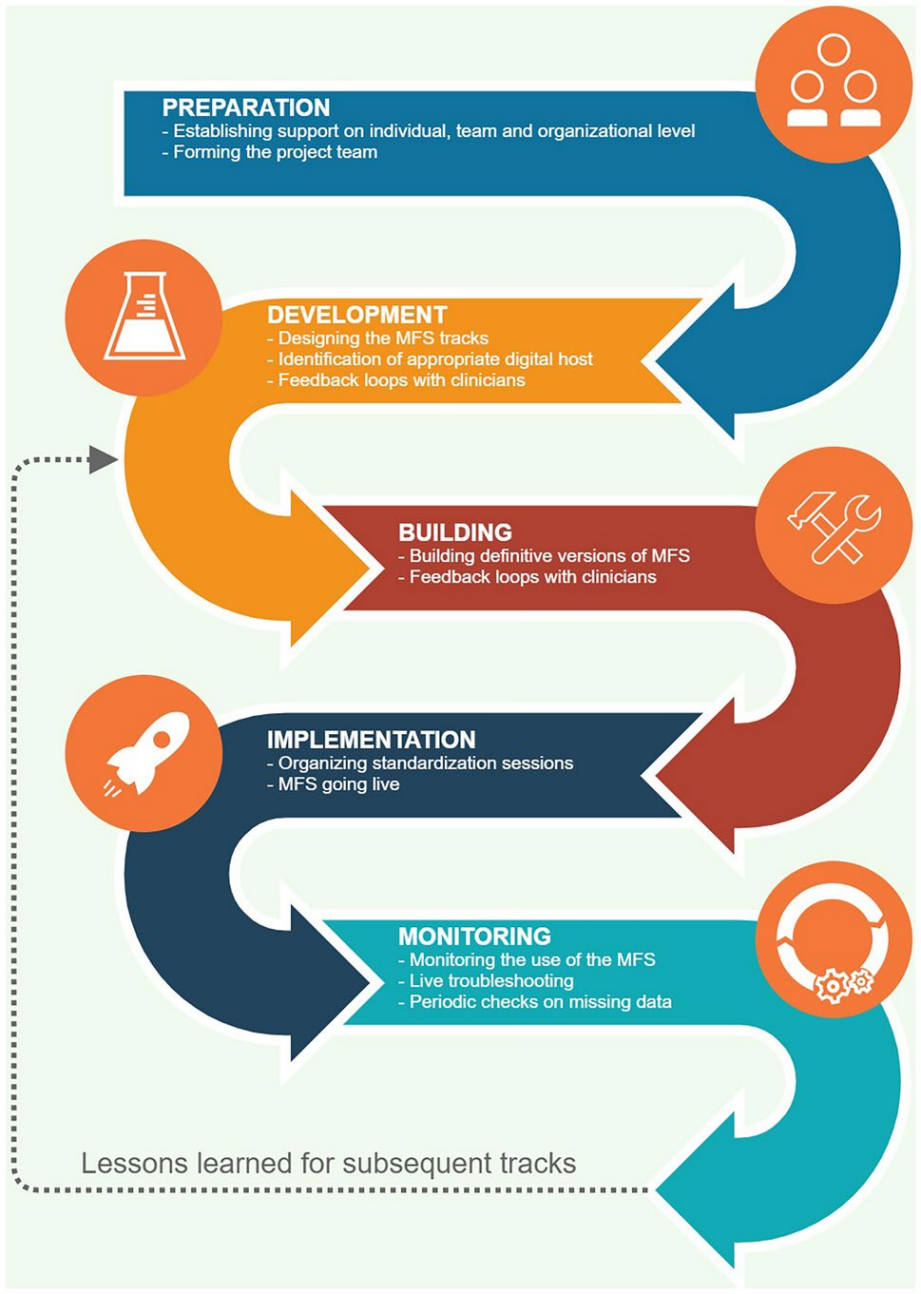
Sequence of steps towards the implementation of the Measurement Feedback System

The MFS was developed to systematically collect, store and visualize clinical information. The development and implementation of the MFS started in September 2018 and was completed in March 2021. The development of the MFS followed a structured stepwise approach, involving the following phases: (1) preparation, (2) development, (3) building, (4) implementation and (5) monitoring, see Fig. 1.

#### Preparation phase

Low organizational priority and support for outcome measurement are recognized challenges to the implementation of structured measurements in clinical practice [27]. Therefore, the project leader started the project by gaining support from the management team, which prioritized development of the MFS as one of the core goals of the organization. This realized a project team consisting of a project leader (part-time) and a project coordinator (full-time) under academic supervision by two principal investigators specializing in Quality Care Programs and Multidisciplinary Team Optimization. Grassroots support was established in addition to organizational support by recruiting one delegate from each discipline to join the MFS development team.

#### Development phase

**Fig. 2:**
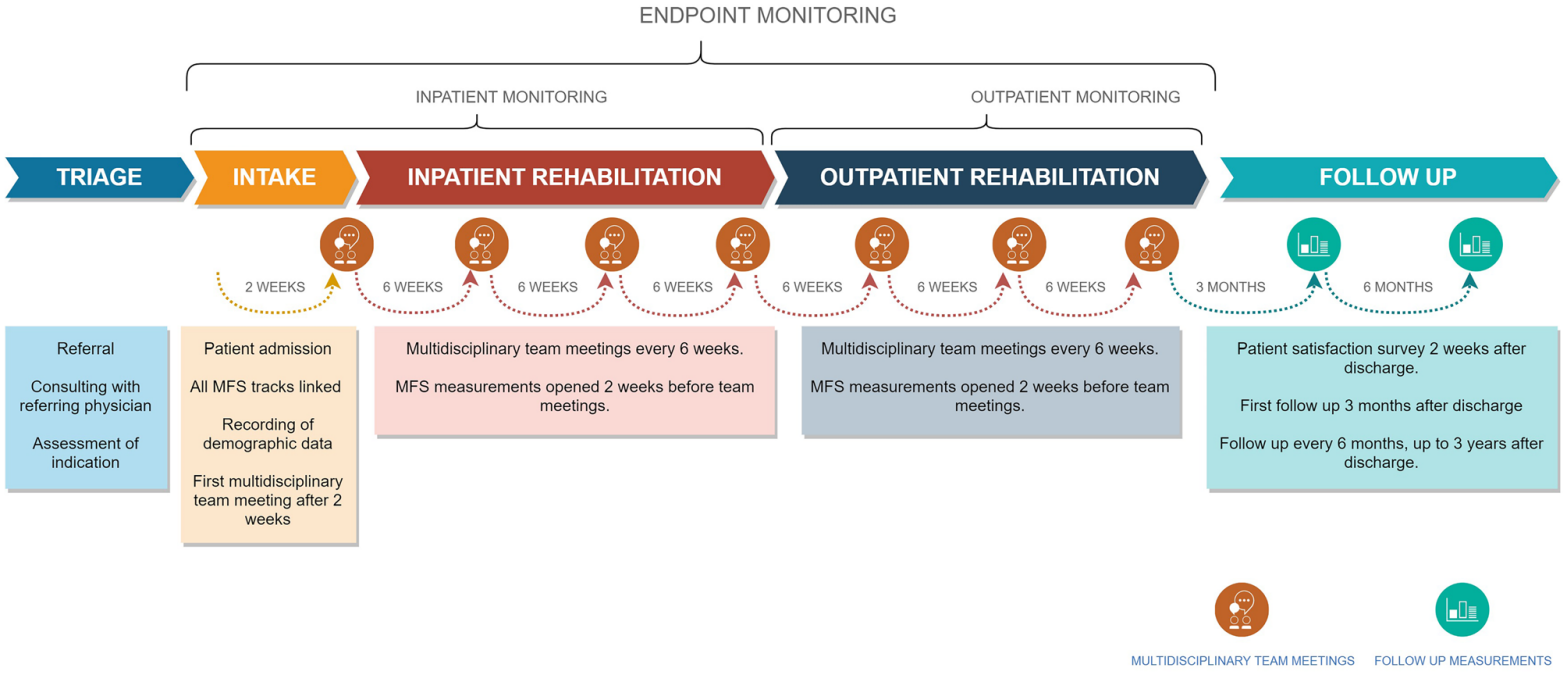
Schematic visualisation of clinical workflow

Clinicians are more positive about the use of measurement instruments when they have a choice over the set of outcome measurements they deem the most relevant to their practice [27]. Consequently, the initial selection of measurements in the MFS was performed by each discipline-specific team. The discipline delegates were instructed to compile a core set of measurements on behalf of their discipline-specific team. The literature indicates that a lack of perceived value of measurements contributes to a reduced chance of their use and sustained adoption [27]. Therefore, the delegates were informed that the core set should (1) provide clinically valuable information; (2) be administered to all patients; (3) cover the discipline's representative domains; and (4) fit in the a-priori defined measurement structure (see Fig. 2). Furthermore, measurements were preferably part of (inter)national guidelines and consensus statements. Measurements with mere scientific relevance were excluded. The project team reviewed the proposed instruments for eligibility, based on the available level of empirical support and their clinimetric properties. If alternative measurements with better clinimetric properties were identified, these were taken into consideration by the discipline delegates, ultimately making a decision while balancing the clinical utility and clinimetric properties of the instrument. After several revisions, this resulted in concept versions of each core set, which were also presented to and agreed upon by the center's scientific advisory board. These core sets were translated into measurement tracks by combining them with the temporal trajectory of neurorehabilitation. Some automated dependencies were built in to avoid illogical measurements being sent out. For example, in the physical therapy track, patients with inability to walk are automatically exempted from measurements requiring the ability to walk, based on their Functional Ambulation Category scale [29] score. The discipline groups were motivated to pilot the use of the core set during a period of three months in order to identify barriers to successful implementation. After fine-tuning of the pilot versions, this led to the definitive core sets.

#### Identification of digital information system

Parallel to the MFS development phase, the electronic infrastructure for MFS data registration and use was identified. A-priori, the following requirements were identified: (1) interface for developing customized registration forms; (2) functionality to orchestrate the timing of measurements; (3) possibility to assign different informants (clinicians, patients, relatives) to measurements; (4) notification function for measurements, with a reminder function; (5) interface for intuitive visualization of measurement results; (6) secured according to local regulations for patient data. The only system that met all requirements was Philips VitalHealth Questionnaire Manager (Vital Health, Ede, the Netherlands), which was therefore contracted to host the MFS.

#### Building phase

The measurements in the definitive core sets were built into the digital environment. First, a registration form was built for each instrument. Then the forms were combined into a measurement track for each discipline. Time points in the measurement tracks were tuned to the planning of multidisciplinary meetings in a fixed 6-week schedule. This ensures that all clinical data is available in the MFS at the time of the multidisciplinary meeting to facilitate decision making. Every time point in a measurement track is initiated by overview of scheduled measurements. To account for exceptional circumstances that may arise in clinical practice, a by-pass was built-in. To by-pass a measurement, the clinician is required to provide a motivation, which is in turn monitored by the project coordinator. The following measurement tracks were developed in overlapping sequential timelines: (1) physical therapy, (2) occupational therapy, (3) speech therapy, (4) neuropsychology, (5) counselling, (6) social work and (7) physical medicine and rehabilitation. Separate questionnaires comprehensively registering demographics characteristics and medical history have been developed and are distributed to the patient's relative within the first two weeks of admission. Furthermore, we have developed structured forms in which referral data, such as injury and acute care characteristics, are entered into the MFS in a structured manner by the project coordinator. The discipline-specific measurement tracks are displayed in Table 1.

**Table 1:** Measurement tracks

**Track**	**Instrument**	**Area of assessment**	**Timepoint**						**Requirements**	**Time (min)**	**Rationale**
			Intake	Inpatient monitoring	Inpatient discharge and/or outpatient intake	Outpatient monitoring	Outpatient discharge	Follow-up			
**Physician**	Demographics, Medical Background, Neuropathology & Acute Treatment Variables	Background	X							120	
	Utrecht Scale for Evaluation of Rehabilitation	Functional independence	X	6w	X					10	NSRM indicator set [40], Post et al. [41]
	Supervision Rating Scale	Supervision	X	6w	X					5	Boake [42]
	Body Mass Index	Nutritional Status	X	6w	X					5	NSRM indicator set [40]
	Short Nutritional Assessment Questionnaire (SNAQ)	Nutritional Status	X		X					5	NSRM indicator set [40]
	Ranchos Los Amigos – Revised: Levels of cognitive functioning	Cognitive Functioning	X	6w	X	6w	X			5	NSRM treatment framework traumatic brain injury [43]
**Physical therapy**	Berg Balance Scale	Balance	X	6w	X	6w	X			15	TBI EDGE [44], KNGF Clinical Practice Guideline for Physical Therapy in patients with stroke [45]
	Motricity Index	Motor impairment	X	6w	X	6w	X			5	StrokEDGE II[46], KNGF Clinical Practice Guideline for Physical Therapy in patients with stroke [45]
	Trunk Control Test	Balance	X	6w	X	6w	X			5	KNGF Clinical Practice Guideline for Physical Therapy in patients with stroke [45]
	Fugl Meyer Assessment – upper extremities	Sensorimotor function	X	6w	X	6w	X			15	KNGF Clinical Practice Guideline for Physical Therapy in patients with stroke [45]
	Erasmus MC modifications to the Nottingham Sensory Assessment	Somatosensory impairments	X	6w	X	6w	X			15	KNGF Clinical Practice Guideline for Physical Therapy in patients with stroke [45], Dutch OT Stroke guideline [47]
	MiniBESTest	Balance	X	6w	X	6w	X			15	TBI EDGE [44]
	Modified Ashworth Scale	Resistance to passive movement	X	6w	X	6w	X			5	KNGF Clinical Practice Guideline for Physical Therapy in patients with stroke [45]
	Functional Ambulation Categories	Gait	X	6w	X	6w	X			5	KNGF Clinical Practice Guideline for Physical Therapy in patients with stroke [45]
	Tecnobody Prokin	Balance (instrumented)	X*	6w*	X*	6w*	X*		*FAC >2	10	Experimental
	10 Meter Walk Test	Gait	X*	6w*	X*	6w*	X*		*FAC >3	5	KNGF Clinical Practice Guideline for Physical Therapy in patients with stroke [45]
	6 Minute Walk Test	Aerobic capacity, gait	X*	6w*	X*	6w*	X*		*FAC >3	10	KNGF Clinical Practice Guideline for Physical Therapy in patients with stroke [45]
	Tecnobody Walkerview	Gait (instrumented)	X*	6w*	X*	6w*	X*		*FAC >4	10	Experimental
	HiMAT (High Level Mobility Test)	Functional mobility	X*	6w*	X*	6w*	X*		*FAC >5	10	TBI EDGE [44]
	Rehabilitation Intensity of Therapy Scale	Intensity of therapy	X	6w	X	6w	X			5	Seel et al. [48]
**Occupational Therapy**	Barthel Index	(Basic) Activities of daily living	X		X		X			10	KNGF Clinical Practice Guideline for Physical Therapy in patients with stroke [45], MDS-ABI [49]
	Canadian Occupational Performance Measure	Occupational performance			X		X			45	Dutch Occupational Therapy Guideline for Stroke [47]
	Action Research Arm Test	Upper extremity functioning	X	6w	X	6w	X			22.5	StrokEDGE II[46], TBI EDGE [44], Dutch Occupational Therapy Guideline for Stroke [47], KNGF Clinical Practice Guideline for Physical Therapy in patients with stroke [45]
	Nine Hole Peg Test	Upper extremity functioning	X*	6w *	X*	6w *	X*		*FMA-UE >5	22.5	Dutch Occupational Therapy Guideline for Stroke [47], KNGF Clinical Practice Guideline for Physical Therapy in patients with stroke [45], StrokEDGE II [46]
	Perceive, Recall, Plan and Perform	Activities of daily living	X		X					45	Dutch Occupational Therapy Guideline for Stroke [47]
	Range of Motion (upper extremity) measured with Tyromotion Pablo	Range of motion (instrumented)	X	6w	X	6w	X			22.5	Experimental
	Grip strength measured with Tyromotion Pablo	Strength (instrumented)	X	6w	X	6w	X			22.5	Experimental
	Rehabilitation Intensity of Therapy Scale	Intensity of therapy	X	6w	X	6w	X			5	Seel et al. [48]
**Speech Therapy**	(shortened) Token Test	Aphasia	X*	3m	X*		X*		*WPTAS >12 + if score >68 every 3m	30	Dutch Logopedic Guideline for the Diagnosis and Treatment of Aphasia [50]
	Screeling	Aphasia	X*	6wk/3m	X*		X*		*WPTAS >12	45	Dutch Logopedic Guideline for the Diagnosis and Treatment of Aphasia [50]
	Dutch Naming Test II	Anomia	X*		X*		X*		*WPTAS >12	50	Dutch Logopedic Guideline for the Diagnosis and Treatment of Aphasia [50]
	Sematic Association Test	Aphasia	X*	3m*	X*	3m*	X*		*WPTAS >12, TT <29 or Screeling < 68	80	Dutch Logopedic Guideline for the Diagnosis and Treatment of Aphasia [50]
	Comprehensive Aphasia Test	Aphasia	X*	3m*	X*	3m*	X*		*WPTAS >12, signs of mild to moderate aphasia	105	Dutch Logopedic Guideline for the Diagnosis and Treatment of Aphasia [50]
	90 ml Water Swallowing Test	Swallowing	X	6w	X	6w	X			15	Dutch Logopedic Guideline on Opharyngeal Dysphagia [51]
	Water Swallow Tests- timed test- Dysphagia limit test	Swallowing	X	6w	X	6w	X			25	Dutch Logopedic Guideline on Opharyngeal Dysphagia [51]
	Diagnostic Instrument Apraxia of Speech	Apraxia of speech	X*		X*		X*		*WPTAS >12, signs of apraxia of speech	120	Aphasia Intervention Scheme of the Dutch Association of Aphasia Therapists [52]
	Dutch Dysarthria Assessment	Dysarthria	X*		X*		X*		*WPTAS >12, signs of dysarthria	50	Aphasia Intervention Scheme of the Dutch Association of Aphasia Therapists [52]
	Radboud Oral Assessment	Oral motor function	X	6w	X	6w	X			15	Kalf & de Swart [53]
	Rehabilitation Intensity of Therapy Scale	Intensity of therapy	X	6w	X	6w	X			5	Seel et al. [48]
**Neuropsychology**	Westmead Post Traumatic Amnesia Scale	Post traumatic amnesia	X	1d*					*WPTAS <12	5	Dutch guideline for neuropsychological assessment in Traumatic brain injury [54]
	Montreal Cognitive Assessment	Cognitive screening	X*		X*		X		*WPTAS =12	11	MDS-ABI [49]
	D-KEFS Trail Making Test	Processing speed, visual attention and cognitive flexibility	X*		X*		X		*WPTAS =12	35	Dutch guideline for neuropsychological assessment in Traumatic brain injury [54], Honan et al. [55]
	D-KEFS Color Word Test	Processing speed, inhibition and cognitive flexibility	X*		X*		X		*WPTAS =12	15	Dutch guideline for neuropsychological assessment in Traumatic brain injury [54]
	Balloon’s Test	Visual inattention	X*		X*		X		*WPTAS =12	15	Lezak et al. [56]
	Wechsler Adult Intelligence Scale Subtests: Similarities, Vocabulary, Matrix Reasoning, Visual Puzzles	Screening of intelligence	X*		X*		X		*WPTAS =12	45	Dutch guideline for neuropsychological assessment in Traumatic brain injury [54], Honan et al. [55] *Nonverbal alternative for Similarities and Vocabulary: Block Design*
	Wechsler Adult Intelligence Scale Subtest: Digit Span	Verbal working memory	X*		X*		X		*WPTAS =12	5	Dutch guideline for neuropsychological assessment in Traumatic brain injury [54], Honan et al. [55] *Nonverbal alternative: Corsi Block Test*
	Auditory Verbal Learning Test (Dutch adaptation)	Episodic verbal memory	X*		X*		X		*WPTAS =12	22	Dutch guideline for neuropsychological assessment in Traumatic brain injury [54]
	Complex Figure of Taylor (modified): Subtest: copy	Visuoconstruction	X*						*WPTAS =12	15	Dutch guideline for neuropsychological assessment in Traumatic brain injury [54]
	Complex Figure of Rey Subtests: copy, immediate recall, delayed recall and recognition	Visuoconstruaction and nonverbal memory			X*		X		*WPTAS =12	15	Dutch guideline for neuropsychological assessment in Traumatic brain injury [54]
	Fluency tests *Category and letter fluency*	Semantic memory and executive functioning	X*		X*		X		*WPTAS =12	15	Dutch guideline for neuropsychological assessment in Traumatic brain injury [54] *Nonverbal alternative: 5-punkt test*
	D2	Attention and processing speed			X*		X		*WPTAS =12	25	Dutch guideline for neuropsychological assessment in Traumatic brain injury [54] *Nonverbal alternatives: WAIS Symbol Search, WAIS Coding*
	Behavioural Assessment of the Dysexecutive Syndrome Subtests: Action Program, Key Search, Zoo Map	Executive functioning	X*		X*		X		*WPTAS =12	30	Dutch guideline for neuropsychological assessment in Traumatic brain injury [54]
	Rivermead Behavioural Memory TestSubtest: Stories	Episodic verbal memory			X*		X		*WPTAS =12	20	Dutch guideline for neuropsychological assessment in Traumatic brain injury [54]
	Location Learning Test	Episodic spatial memory	X*		X*		X		*WPTAS =12	40	Dutch guideline for neuropsychological assessment in Traumatic brain injury [54]
	Tower of London Test	Executive functioning	X*		X*		X		*WPTAS =12	20	Dutch guideline for neuropsychological assessment in Traumatic brain injury [54]
	Checklist for Cognitive and Emotional Consequence of Stroke – self-rating	Cognition, emotion	X*		X*		X		*WPTAS =12	10	van Heugten et al. [57]
	Rehabilitation Intensity of Therapy Scale	Intensity of therapy	X	6w	X	6w	X			5	Seel et al. [48]
**Counseling**	Hospital Depression and Anxiety Scale	Depression and anxiety	X*		X*		X		* WPTAS =12	10	MDS-ABI[49], Honan et al. [55]
	Rosenberg Self-Esteem Scale	Self-esteem	X*		X*		X		* WPTAS =12	5	Honan et al. [55]
	Coping Inventory for Stressful Situations	Coping	X*		X*		X		* WPTAS =12	10	Brands et al. [58]
	Checklist Individual Strength	Fatigue	X*		X*		X		* WPTAS =12	10	Worm-Smeitink et al. [59]
	Checklist for Cognitive and Emotional Consequence of Stroke – proxy rating	Cognition, emotion								10	van Heugten et al. [57]
	Neuropsychiatric Inventory Questionnaire	Behavior								10	NSRM Dutch Guideline Neuropsychiatric consequences after NAH in adults [60]
	ALCOS-12 General Competence Scale	Self efficacy	X*		X*		X		* WPTAS =12	5	Bosscher and Smit [61]
	Rehabilitation Intensity of Therapy Scale	Intensity of therapy	X	6w	X	6w	X			5	Seel et al. [48]
**Social Work**	Caregiver Strain Index	Caregiver strain	X	6w	X	6w	X	X		5	Dutch Occupational Therapy Guideline for Stroke [47]
	Self-Sufficiency Matrix	Self-sufficiency			X	3m	X	X		15	experimental
	Quality of Life after Brain Injury (QOLIBRI)	Quality of life					X	X		10	TBI EDGE [44], StrokEDGE II [46]
	Utrecht Scale for Evaluation of Rehabilitation-Participation	Participation			X	6w	X	X		10	MDS-ABI [49]
	De Jong Gierveld Loneliness Scale	Loneliness			X	6w	X	X		5	De Jong Gierveld and van Tilburg [62]
	Rehabilitation Intensity of Therapy Scale	Intensity of therapy	X	6w	X	6w	X			5	Seel et al. [48]

*6w* 6 weeks, *3m* 3 months, *FMA-UE* Fugl Meyer Assessment – upper extremities, *TT* (shortened) Token Test, *WPTAS* Westmead Post Traumatic Amnesia Scale

* indicates a requirement before administering

#### Implementation phase

After completion of a measurement track in the digital environment, standardization sessions were scheduled, together with the project team and all members of the involved discipline. These sessions were intended to stimulate standardization of measurements across respondents and used available manualized instructions, if available. Accordingly, each measurement procedure was explained and demonstrated in a test procedure in front of the whole discipline group, after which perceived differences in the administration procedure were discussed and harmonized in a meeting record and added to the instruction of the measurement form in the MFS. In the same session, clinicians were introduced and familiarized with the use of the electronic MFS hosting environment. Final adjustments to the measurement tracks were made in the two weeks following the standardization session, after which the measurement track went live. As limited time has been described as a significant factor influencing successful implementation of outcome measurements [27, 30], we constructed an overview of time needed to complete and analyze the measurements, which was subsequently approved by the management team. As a result, clinicians can allocate the necessary time in their schedule for MFS activities, lowering the risk that MFS is omitted due to time pressure arising from other clinical activities.

#### Monitoring phase

On admission of a patient, all measurement tracks are manually linked to the assigned clinicians. E-mail notifications are sent to the clinicians automatically two weeks prior to the interdisciplinary team meetings, and reminder notifications are sent before the deadline. Clinicians can access the measurement forms via a link in the e-mail notification or through Questionnaire Manager’s main portal. An extract of the database is made weekly, for which we have built an automatic processing pipeline that produces monitoring summaries including completeness of registrations on level of (i) the center as a whole; (ii) treatment disciplines; and (iii) individual clinicians.

#### Data collection and security

All data are collected using Philips VitalHealth Questionnaire Manager (Vital Health, Ede, the Netherlands). Questionnaire Manager is a secure web-based application for conducting diagnostic, quality and effect measurements within healthcare. Data in Questionnaire Manager are protected at the level required for electronic patient records to ensure data safety, like monitoring of all activity on the platform and two-factor authentication for login. The system complies with the international standards ISO 27001, ISO 13485 and NEN 7510. Patients and families are asked for permission to use their pseudonymized data for scientific research at admission. Otherwise, patient records are solely used for healthcare purposes as described in the Dutch Medical Treatment Contracts Act (WGBO) [31].

#### Data collected

At time of writing, 124 patients have been registered on the MFS platform, providing an average of more than 200 new measurements per week. Currently, more than 15,000 clinical measurements were captured in the MFS. The current overall completion rate of measurements is 86,4%. A summary of completeness per discipline is enclosed in the Supplements (efigure 4).

#### Patient dashboards

**Fig. 3:**
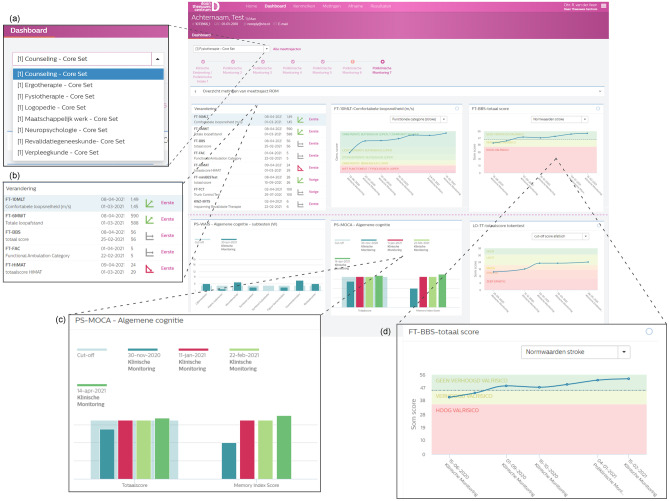
Screenshot of the platform with (**a**) a possibility to toggle between different trajectories. (**b**) An overview of the change. (**c**) An example of bar graph, in light blue a cutoff score. (**d**) An example of line graph, with normative data in color

The scores of all measurements are visualized in individual patient dashboards that are accessible for all clinicians involved in treatment. The user is able to toggle between the different measurement tracks and different time points. Figure 3 shows a screenshot of the digital platform and cutouts of various sections of the dashboard. Clinically significant change can also be indicated on the dashboard when the necessary information about the measurement instrument's reliability is known and registered in the system. The evolution of measurements over time is visualized in line graphs (for overall scores) or bar graphs (for subscale scores). If available, normative data is displayed as a reference in the graphs, facilitating better understanding and interpretation of measurements between disciplines. The platform makes it possible to generate automated PDF reports for patients. Also, we plan to launch a secure portal for patients and their families to see their individual progress over time for data that is disclosed by the clinician.

## Discussion

In this study, we described the development and implementation of a MFS for neurorehabilitation after severe ABI. As noted, the MFS has contributed to structured clinical assessment based on multidisciplinary consensus, comprehensive progress monitoring for each discipline, multidisciplinary collaboration through cross-discipline access to patient dashboards and shared decision making based on a rich information source. Moreover, accumulation of MFS data at the group level facilitates care evaluation and innovation and may contribute to the shift towards precision medicine in neurorehabilitation.

### Challenging and success factors

The MFS has been implemented successfully, as indicated by the relatively high completion rate as compared to other MFS implementations ranging between 47–70% [32]. Nevertheless, we also encountered several challenges. We discovered that the rationale for structured clinical assessment as part of the MFS does not stay top of mind in daily clinical practice. The clinician’s view frequently shifted towards practicality and relevance of measurements for particular individual patients, while the overarching value of MFS regularly faded more into the background. Therefore, additional briefings for the clinical staff proved to be needed more than initially anticipated, and we realize that this is probably an ongoing process. Moreover, better monitoring of pilot phase execution could have prevented the need for late amendments to measurement tracks that were discovered shortly after actual implementation.

The project team also detected key success factors in the development and implementation. According to Douglas et al., one of the key principles for sustainable adoption and implementation of a MFS is an emphasis on the integration with clinical values and workflow [33]. This requires high flexibility in the design of the electronic environment that is used to host the MFS. Bickman et al. report that the most prominent obstacle to adoption of their MFS was the occurrence of technical issues [32]. Indeed, we identified autonomy in the build of the MFS platform as a key success factor, allowing us to fit the MFS to our local work flow while also troubleshoot technical issues directly. We consider prioritization of this project on the organizational level as the most crucial facilitator in the process. This is consistent with literature indicating that organizational factors play an important role in the use of evidence based practice in general [34] and outcome measurement practices in particular [35]. We experienced a constructive attitude among clinicians and a high level of engagement in the development of the new work process. Employee turnover, on the other hand, can result in a future lack of knowledge and/or involvement. This emphasizes the importance of ongoing support, instruction, and never-ending "buy-in talks." Likewise, even after successful implementation, organizational priority will continue to be an important factor in MFS success. Since a rate of missing data of more than 15% is problematic for the application of various statistical techniques [36], we strive for a completion rate of at least 90% in the forthcoming period. Regarding the clinimetric properties of the included measuring instruments, statistically combining the data (e.g., using principal component analysis) from multiple measurements, also with availability of repeated measurements over time, may increase the robustness of measurements from instruments with less favourable clinimetric properties.

### Future directions for MFS data: the Health Intelligence Program

With structured clinical assessment in place, accumulation of MFS data at the group level is considered to present a valuable source for additional higher goals, which have been formulated in the Health Intelligence Program of the Daan Theeuwes Center. The first goal is to execute continuous care evaluation and innovation. Therefore, our project team is currently working on a standardized processing pipeline for analysis and visualization of MFS data at the group level. The findings will be used as input for periodic care evaluation sessions at various levels of the organization, with the goal of identifying innovations that can further improve care quality. We envision several clinical applications of MFS data analysis. For example, we aim to develop reference values for recovery trajectories in our center. This allows individual bench-marking allowing identification of patients with relatively little recovery progress. For such patients, we hope to implement an internal second-opinion cycle, by presenting the treatment plan to a parallel interdisciplinary team.

The second goal of the Health Intelligence Program is to contribute to the shift towards precision medicine in neurorehabilitation. Recently it has been suggested that the field of ABI research should move away from underpowered, case–control designs that are limited by the heterogeneity of the ABI population [15]. Instead we should seek to use methods that can take into account the inherent heterogeneity, so that differences between patients can be used to better understand and predict outcomes and rehabilitation potential. Several promising approaches are emerging, such as using machine learning algorithms for clinical prediction modelling [37]. We are already starting to see these approaches emerge in stroke rehabilitation research [38, 39], implying the field might be on the brink of advancing toward data-driven precision rehabilitation medicine. We hope to use MFS data to improve the prediction of neurorehabilitation outcome, utilizing the dimensionality of the data to provide a more personalized prognosis. First-tier analyses will concentrate on using the data with the highest availability (i.e. intake data), to better understand heterogeneity in patient functioning. We intend to use data-driven approaches to cluster measures of patient functioning into domains and cluster patients into subgroups with comparable function profiles. This will allow for a more thorough and systematic understanding of patient differences across domains of functioning. As more data becomes available, the focus of our work will shift towards the development of personalized outcome prediction models aimed at treatment outcomes that are most important to the patient and rehabilitation team (e.g. able to walk independently, able to return to work, etc.). One of the most ambitious goals of our current research agenda is to create a tool that can use all available MFS data from a given patient to model individual recovery trajectories. Such a tool could provide chance estimations for the occurrence of meaningful change in selected outcomes within a pre-defined timeframe (e.g. the upcoming treatment period). A decision support system of this type could provide the rehabilitation team with additional quantitative input for the decision whether or not to continue the rehabilitation treatment.

## Conclusions

This study describes the process of successful development and implementation of a MFS in an interdisciplinary neurorehabilitation setting. We have shown that structured clinical assessment and feedback is realistic and feasible in the context of neurorehabilitation after severe ABI, while considering key success factors. We consider the described approach transplantable to other settings that aim to improve the quality of care for complex patient populations.

## Supplementary Information

Below is the link to the electronic supplementary material.

Supplementary file1 (JPG 1597 KB)

## Declarations

### Research involving human and animal participants

This article does not contain any studies with human participants performed by any of the authors.
