# Supplementary figures and images for: Measurement Feedback System for Intensive Neurorehabilitation after Severe Acquired Brain Injury
Source: J Med Syst. 2022 Apr 4;46(5):24. doi: 10.1007/s10916-022-01809-z (PMC8979932; doi:10.1007/s10916-022-01809-z)

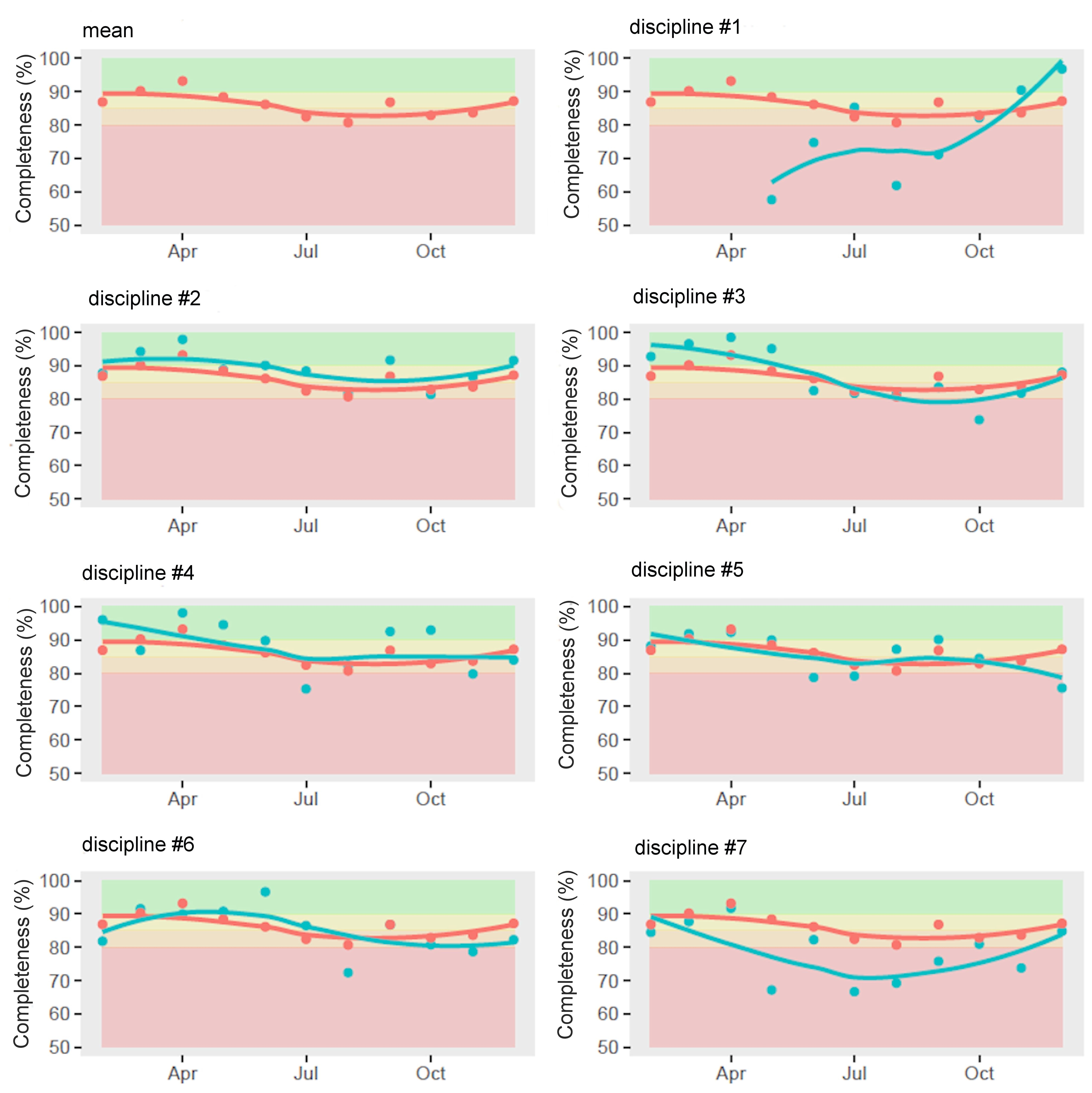

Supplement: Supplementary file 1 — Supplementary file1 (JPG 1597 KB) [file 10916_2022_1809_MOESM1_ESM.jpg]
